# Isoform-resolved 14-3-3/YWHA networks in colorectal cancer: phospho-adaptor mechanisms, biomarker contexts and therapeutic opportunities

**DOI:** 10.3389/fonc.2026.1906093

**Published:** 2026-08-19

**Authors:** Qian Liu, Rudong Li, Wei Ye, Zhipeng Zhao

**Affiliations:** 1 Department of Respiratory and Critical Care Medicine, Shandong Provincial Third Hospital, Shandong University, Jinan, Shandong, China; 2 Department of Gastrointestinal Nutrition and Hernia Surgery, The Second Hospital of Jilin University, Changchun, Jilin, China

**Keywords:** 14-3-3 proteins, colorectal cancer, extracellular vesicles, immune exhaustion, patient-derived organoids, phosphorylation-dependent adaptor proteins, YWHA

## Abstract

YWHA/14-3-3 proteins are conserved phosphoserine/phosphothreonine-binding adaptors that coordinate signaling-complex assembly, subcellular localization, stress responses and cell-state transitions. Although often discussed as a single adaptor family, colorectal cancer (CRC) studies suggest that individual YWHA isoforms act through distinct clients, RNA-associated layers and tumor-state contexts. In this narrative and mechanistic review, we synthesized PubMed-indexed CRC literature through 18 May 2026 and appraised CRC-relevant mechanistic modules using an explicit evidence-maturity rubric developed by the authors distinct from formal GRADE assessment. We propose an isoform–client–context framework for interpreting 14-3-3 biology in CRC. YWHAG has been linked to CTTN-dependent Wnt/β-catenin activation, whereas YWHAE/14-3-3ϵ protein has been associated with extracellular-vesicle secretion and EV-associated β-catenin/Wnt outputs. Separately, the YWHAE-encoded lncRNA, hereafter referred to as YWHAE lncRNA, has been proposed to activate KRAS/ERK and PI3K/AKT signaling through a competing endogenous RNA mechanism. YWHAZ participates in epithelial–mesenchymal and G2/M transitions through miR-1-3p- and TRIP13-associated mechanisms. YWHAB supports PIK3R2-dependent PI3K/AKT signaling, whereas YWHAH connects NAT10/ac4C regulation, CD8+ T-cell exhaustion and MAPK/ERK-dependent autophagy-associated invasion. SFN illustrates context-dependent tumor-suppressive or stress-adaptive functions. Overall, total 14-3-3 abundance is insufficient for mechanistic or translational interpretation. Interactomics, phosphoproteomics, spatial profiling, extracellular-vesicle analysis and patient-derived models should be used to prioritize YWHA-dependent candidate mechanisms for functional validation. These modules should currently be regarded as testable biomarker and therapeutic hypotheses, not as clinically validated CRC biomarkers or targets.

## 1 Introduction

Colorectal cancer (CRC) remains one of the most common malignancies worldwide and continues to cause substantial cancer-related mortality (1, 2). Its biological behavior is shaped by canonical driver alterations, including APC/Wnt dysregulation, RAS/MAPK activation, PI3K pathway signaling, TP53 disruption, SMAD4/TGF-β pathway alteration, chromosomal instability, microsatellite instability and tumor-microenvironment remodeling (3, 4). Large-scale proteogenomic analysis has also shown that transcript abundance alone does not fully capture protein-level pathway activity or therapeutic vulnerabilities, a point that is especially relevant for adaptor proteins whose function depends on protein complexes and post-translational modifications (5).

CRC progression is driven not only by classical oncogenes and tumor suppressors, but also by adaptor and scaffold proteins that organize signaling outputs. These proteins translate phosphorylation, localization and stress cues into functional pathway states. Wnt/β-catenin signaling, extracellular vesicle (EV)-mediated communication, epithelial-mesenchymal transition (EMT), PI3K/AKT survival signaling, autophagy, unfolded protein response (UPR), immune escape and therapy-induced adaptation all require dynamic protein complexes (6–10). CRC is therefore a particularly suitable disease context in which to examine phosphorylation-dependent adaptor networks.

YWHA/14-3-3 proteins are among the best-characterized phosphoserine/phosphothreonine-binding adaptor proteins (11–16). The mammalian family includes YWHAB/14-3-3β, YWHAE/14-3-3ϵ, YWHAG/14-3-3γ, YWHAH/14-3-3η, YWHAQ/14-3-3θ/τ, YWHAZ/14-3-3ζ and SFN/14-3-3σ. These isoforms share a conserved dimeric architecture and frequently bind phosphorylated clients, thereby altering client localization, stability, enzymatic activity, dimerization, complex formation or degradation (11–16). Historically, many cancer studies have discussed them collectively as “14-3-3 proteins”. However, family-level terminology can obscure biologically meaningful isoform-specific mechanisms.

Recent CRC studies make an isoform-resolved interpretation increasingly important. YWHAG has been identified as a CTTN-dependent Wnt/β-catenin amplifier in CRC (17). YWHAE regulates EV secretion and Wnt/β-catenin signaling, and the YWHAE locus encodes a long non-coding RNA that activates KRAS/ERK and PI3K/AKT pathways through miRNA sponging (18, 19). YWHAZ participates in EMT and G2/M transition through miRNA- and TRIP13-associated mechanisms (20–23). YWHAB supports PIK3R2-dependent PI3K/AKT signaling and has been included in chromosome 20q-associated progression signatures (24–26). YWHAH has been linked to NAT10/ac4C-mediated CD8+ T-cell exhaustion and MAPK/ERK-dependent autophagy-associated invasion (27, 28). SFN/14-3-3σ, in contrast, illustrates context-dependent duality (29–37).

**Figure 1:**
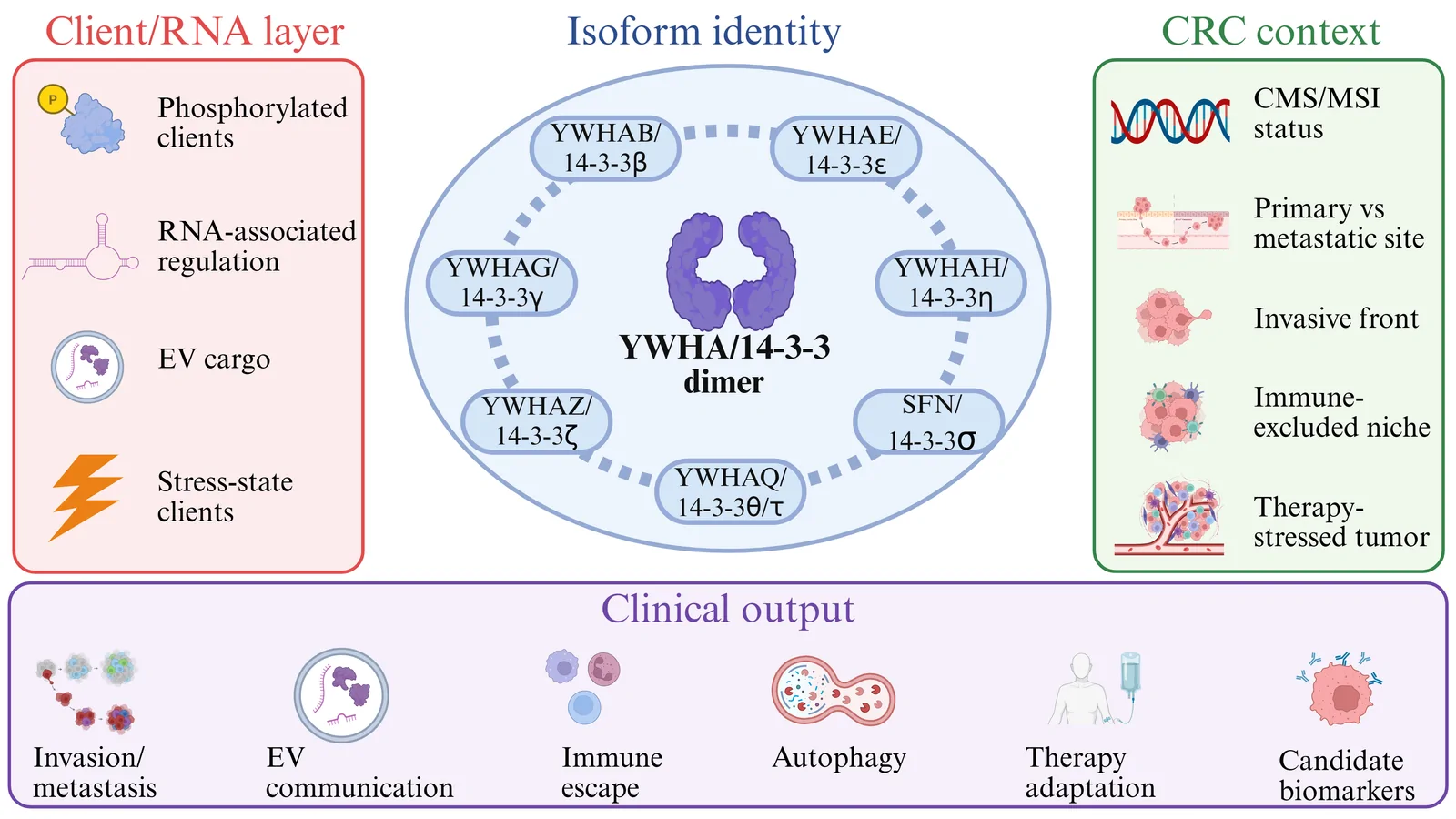
Isoform–client–context framework of YWHA signaling in CRC.

In this review, we synthesize CRC-specific evidence for YWHA/14-3-3 isoforms and organize the field around an isoform–client–context framework. Rather than classifying 14-3-3 proteins as uniformly oncogenic or tumor suppressive, we examine how individual isoforms engage distinct phosphorylated clients, RNA-associated regulatory layers, subcellular compartments and tumor states. We further consider how these modules may inform biomarker development, spatial and extracellular-vesicle profiling, patient-derived validation models and interaction-guided therapeutic hypotheses. The central premise of this framework is that the biologically and clinically relevant unit is not total 14-3-3 abundance, but a context-defined active YWHA module. Such a module is jointly determined by isoform identity, the relevant client or RNA/EV-associated layer, the phosphorylation- or complex-dependent interaction, the subcellular or spatial compartment, the CRC molecular state, and the resulting functional or clinical output. This framework is summarized in **Figure 1**.

## 2 Literature search strategy and evidence classification

PubMed was searched up to 18 May 2026 using combinations of YWHA/14-3-3 gene or protein names and CRC-related terms. The YWHA/14-3-3 terms included “YWHA”, “14-3-3”, “YWHAG”, “YWHAE”, “YWHAZ”, “YWHAB”, “YWHAH”, “YWHAQ”, “SFN” and “stratifin”. The disease terms included “colorectal cancer”, “colon cancer”, “rectal cancer”, “colorectal carcinoma”, “colon carcinoma” and “rectal carcinoma”. A representative search strategy was: (“YWHA” OR “14-3-3” OR “YWHAG” OR “YWHAE” OR “YWHAZ” OR “YWHAB” OR “YWHAH” OR “YWHAQ” OR “SFN” OR “stratifin”) AND (“colorectal cancer” OR “colon cancer” OR “rectal cancer” OR “colorectal carcinoma” OR “colon carcinoma” OR “rectal carcinoma”). CRC-specific mechanistic studies were prioritized. Non-CRC studies were included only when they provided structural, therapeutic, technical or general 14-3-3 mechanistic context needed to interpret CRC findings.

This article is a narrative and mechanistic review rather than a systematic review or meta-analysis. Accordingly, no PRISMA-based screening workflow, formal risk-of-bias assessment or quantitative evidence synthesis was performed. Bibliographic information was checked against PubMed/NCBI and publisher records where possible. To improve transparency and limit overinterpretation, we applied an author-developed evidence-maturity framework that is distinct from formal GRADE assessment.

The unit of appraisal was the individual CRC-relevant mechanistic module rather than an entire YWHA isoform or a single publication. Each module was evaluated across six domains: (i) evidence from human CRC tissues, clinically annotated cohorts or patient-derived models; (ii) direct support for the proposed molecular relationship, such as protein interaction, phosphorylation-dependent binding, transcriptional regulation, RNA-target validation or extracellular-vesicle cargo analysis; (iii) functional perturbation of the YWHA isoform and/or its proposed client or regulatory partner; (iv) causal reinforcement through rescue, epistasis or phosphosite-mutant experiments; (v) functional validation in an *in vivo* CRC tumor, progression or metastasis model; and (vi) independent, spatially resolved or therapy-response validation.

Each module was independently reassessed by two authors according to these criteria, and disagreements were resolved by consensus with a third author. A category was assigned only when the minimum criteria for that level were satisfied. When the available evidence was heterogeneous or fell between two categories, the lower category was retained and the principal missing evidence was specified in **Table 1**.

**Table 1:** Module-level evidence maturity of YWHA-related mechanisms in CRC.

Module	Key CRC-specific evidence	Evidence types and models	Evidence maturity category	Principal evidence gap
YWHAG–CTTN–Wnt/β-catenin	YWHAG is upregulated in CRC and associated with poor prognosis. YWHAG interacts with CTTN and promotes proliferation, migration, invasion and Wnt/β-catenin signaling (17).	CRC tissues, CRC cell lines, protein-interaction and pathway assays.	B	Independent cohorts, *in vivo* causal validation and spatial analysis at invasive fronts or metastatic sites are lacking.
YWHAE protein–EV/Wnt	YWHAE silencing inhibits CRC-cell proliferation and migration, reduces EV secretion and decreases EV-associated β-catenin and Wnt-related signaling (18).	CRC tissues, CRC cell lines, EV-secretion and cargo assays.	B	Direct YWHAE client interactions, functional cargo transfer to recipient cells, *in vivo* validation and liquid-biopsy replication remain unproven.
YWHAE lncRNA–miR-323a-3p/miR-532-5p–KRAS/ERK/PI3K/AKT	The YWHAE lncRNA competes with miR-323a-3p and miR-532-5p and promotes KRAS/ERK and PI3K/AKT signaling (19).	Small CRC tissue set, public transcriptomic datasets and CRC cell lines; mechanistic assays mainly in HCT116 cells.	C	Evidence relies predominantly on one cell-line model, with limited cohort breadth and no patient-derived or *in vivo* validation.
miR-1-3p–YWHAZ–EMT	miR-1-3p directly targets YWHAZ and suppresses CRC-cell proliferation, metastatic phenotypes and EMT-related changes (20).	CRC tissues and cell lines, miRNA-target validation and functional assays.	B	Independent clinical validation, spatial localization and therapy-response relevance remain limited.
TRIP13–YWHAZ–G2/M transition and EMT	TRIP13 interacts with YWHAZ and promotes CRC proliferation, G2/M transition, EMT, invasion and tumor growth (21).	CRC tissues and cell lines, protein-interaction assays and TRIP13-directed *in vivo* models.	B	YWHAZ-specific rescue, *in vivo* dependency and contribution to the response to TRIP13 inhibition have not been established.
YWHAB–PIK3R2–PI3K/AKT	YWHAB binds PIK3R2; YWHAB depletion suppresses PI3K/AKT signaling and malignant phenotypes, whereas PIK3R2 overexpression provides functional rescue (24).	Colon cancer cell lines, protein-interaction, pathway and rescue assays.	C	Direct patient-tissue evidence, patient-derived models and *in vivo* validation are lacking.
YWHAB–chromosome 20q progression signature	YWHAB has been included in chromosome-20q-associated CRC progression signatures (25).	Genomic and clinical-cohort association analyses.	C	The independent contribution of YWHAB at the protein and mechanistic levels has not been demonstrated.
NAT10/ac4C–YWHAH–CD8+ T-cell exhaustion	NAT10-mediated ac4C regulation increases YWHAH stability and is associated with impaired CD8+ T-cell activity, immune escape and adverse outcome (27).	Human CRC data, CRC cell models, RNA-modification and stability assays, CRC cell–CD8+ T-cell coculture.	B	Immune-competent tumor models, spatial immune validation and immune-checkpoint-therapy response data are lacking.
YWHAH–MAPK/ERK–autophagy-associated invasion	YWHAH is associated with adverse clinicopathological features and promotes migration and invasion through MAPK/ERK-associated autophagy regulation (28).	CRC tissues, CRC cell lines, pathway and functional assays.	B	Autophagic-flux direction, causal rescue, *in vivo* validation and clinical predictive value remain insufficiently defined.
SFN loss–intestinal tumorigenesis and epithelial destabilization	SFN loss is linked to adverse CRC features and promotes adenoma formation and epithelial destabilization in intestinal tumor models (31).	Human CRC tissue and clinical-outcome associations; genetically modified intestinal tumor models and transcriptomic analyses.	B	A single defined SFN client-dependent mechanism and corresponding rescue or epistasis experiment have not been established across the complete module.
SFN–NEDD4L–HIF-1α	SFN promotes the interaction of S448-phosphorylated NEDD4L with HIF-1α, enhancing HIF-1α ubiquitination and proteasomal degradation. The module suppresses hypoxia-associated angiogenesis and metastasis and enhances the antitumor effect of bevacizumab (32).	Human CRC evidence, direct interaction and phosphorylation-dependent assays, ubiquitination studies, functional perturbation and CRC xenograft treatment models.	A	Independent prospective clinical validation and spatial assessment of the active SFN–NEDD4L–HIF-1α complex are still required.
TGF-β/SMAD4–SFN–TFEB	SMAD4 directly induces SFN; SFN is required for SMAD4-induced mesenchymal–epithelial transition and autophagy inhibition and binds TFEB in a Ser211 phosphorylation-dependent manner. SFN knockdown and TFEB-S211A provide epistasis and rescue evidence (34).	Human CRC cell lines, patient-derived CRC organoids, transcriptional and protein-interaction assays, phosphosite-mutant rescue, mouse intestinal epithelium and adenoma transcriptomic analysis.	B	Direct patient-cohort validation and functional testing of the complete axis in an *in vivo* CRC tumor, invasion or metastasis model are lacking.
SFN–YY1–UPR and therapy resistance	SFN binds YY1 and restricts its nuclear localization, thereby maintaining UPR-gene expression, tumor growth and resistance to 5-fluorouracil. SFN knockout and re-expression, YY1-binding mutants, UPR perturbation and xenograft experiments support causality (33).	Human CRC datasets, multiple CRC cell models, direct interaction and mutant assays, genetic and pharmacological rescue, chemotherapy assays and CRC xenografts.	A	Independent treatment-annotated patient cohorts and prospective validation as a predictive chemotherapy biomarker are lacking.
SFN–tenascin-C/invasive-front phenotype	SFN overexpression was associated with poor survival in CRC (35). A separate study linked SFN expression in budding tumor cells to tenascin-C-dependent migration (36).	Patient CRC tissues, clinicopathological association and limited cell-migration assays.	C	A defined phosphorylation-dependent client complex, causal rescue and *in vivo* invasive-front validation are lacking.
EV-associated SFN–perineural invasion	Serum EV-associated SFN has been associated with perineural invasion and adverse prognosis in stage II CRC (37).	Clinical serum-EV cohort and outcome association.	C	Independent cohorts, standardized EV characterization, tissue–EV concordance and functional causality remain unvalidated.
YWHAQ-associated CRC mechanisms	No sufficiently developed CRC-specific YWHAQ mechanism was identified within the reviewed literature.	Indirect or discovery-level evidence.	D	Isoform-specific interactomics, phosphoproteomics, proximity labeling and functional validation are required.

Evidence-maturity criteria are defined in Section 2. Categories are not formal GRADE ratings and do not indicate clinical readiness.

Category A was reserved for modules supported by human CRC evidence, a directly demonstrated molecular mechanism, functional perturbation, causal rescue or epistasis, and functional validation in an *in vivo* CRC tumor or progression model. Category B indicated human CRC, patient-derived or clinically annotated evidence together with direct mechanistic and functional support, but with incomplete *in vivo* tumor validation, independent clinical replication, spatial validation or therapy-response evidence. Category C indicated CRC-specific evidence derived mainly from cell-line studies, database analyses, a limited or single cohort, or an incompletely established causal chain. Category D indicated indirect, discovery-level or evidence-gap status without a sufficiently developed CRC-specific mechanism.

These categories describe the completeness and maturity of the CRC-specific evidence chain. They do not represent formal GRADE ratings, risk-of-bias judgments, effect-size estimates or levels of clinical readiness. A lower category does not imply that a module is biologically unimportant; rather, it identifies the major experimental or clinical validation steps that remain necessary. The resulting module-level classifications are summarized in **Table 1**.

## 3 Phospho-adaptor biology of YWHA/14-3-3 proteins

The core molecular function of 14-3-3 proteins is phosphorylation-dependent client recognition. 14-3-3 dimers usually bind phosphoserine- or phosphothreonine-containing motifs and influence client-protein stability, localization, enzymatic activity, scaffold formation or interaction with other proteins (11–15). Structural and computational studies show that the binding groove can accommodate diverse phosphorylated peptides and that local peptide sequence, conformational flexibility and flanking structural context influence binding specificity (14, 15).

Isoform specificity may arise at several levels. First, individual isoforms differ in tissue expression, subcellular distribution, inducibility and stress-response context (14, 16). Second, 14-3-3 proteins form homodimers and heterodimers, potentially creating different client-binding geometries. Third, isoforms may regulate the same signaling pathway at different molecular entry points. A mitotic checkpoint study showed that 14-3-3ϵ and 14-3-3γ interact with CDC25C, whereas 14-3-3σ controls mitotic entry through a CDC25C-independent mechanism (38). This finding illustrates functional non-equivalence within the family.

These molecular principles have direct translational implications. Because 14-3-3 proteins interact with many clients, pan-family inhibition could disrupt both oncogenic and protective complexes. Current drug-discovery efforts increasingly focus on selective modulation of defined protein–protein interactions, including stabilization of selected 14-3-3-client complexes rather than nonspecific pan-family blockade (13, 39–41). In CRC, total YWHA expression is therefore unlikely to be a sufficient biomarker; the actionable unit is a context-defined active YWHA module.

## 4 Isoform-resolved YWHA networks in CRC

### 4.1 Family-level expression and compartment-aware interpretation

An early comparative study of sporadic colorectal adenocarcinoma examined all seven 14-3-3 isoforms and reported decreased expression of σ, η and ζ in adenocarcinomas compared with normal tissue, with methylation changes affecting σ and η promoters (42). This study established that the family cannot be interpreted as uniformly upregulated or uniformly oncogenic in CRC. It also indicated that epigenetic regulation and isoform identity are both relevant.

Proteomic and microenvironmental studies further support compartment-aware interpretation. CRC cell-line proteomics identified 14-3-3 family-related expression changes associated with secretion, metastasis-associated serum/ascites activity and EMT-related processes (43). Another CRC study suggested that 14-3-3ζ/δ and ezrin may participate in motility and invasion of polyploid giant cancer cells and their daughter cells (44). These findings are less isoform-resolved than recent mechanistic studies, but they reinforce the broader concept that YWHA proteins participate in cytoskeletal remodeling, invasion and tumor progression.

### 4.2 YWHAG/14-3-3γ: CTTN-dependent Wnt activation

YWHAG/14-3-3γ represents one of the strongest CRC-specific examples of isoform-resolved YWHA signaling. A 2024 study identified YWHAG as a significantly upregulated YWHA family member in CRC tissues and associated high YWHAG expression with poor prognosis (17). Functional experiments showed that YWHAG promoted CRC cell proliferation, migration and invasion, supporting a direct tumor-promoting role.

Mechanistically, RNA sequencing linked YWHAG to Wnt and EMT-related pathways. CTTN, also known as cortactin, was identified as a YWHAG-associated protein, and the YWHAG-CTTN interaction mediated tumor-promoting activity through activation of Wnt/β-catenin signaling (17). This finding is important because Wnt/β-catenin activation is central to CRC initiation and progression, but downstream amplifier mechanisms remain important for invasion, tumor budding and treatment adaptation (6–8). YWHAG may therefore operate not as a primary initiating Wnt lesion but as an adaptor-dependent amplifier of Wnt-associated invasive signaling.

A key translational question is whether YWHAG identifies a clinically meaningful Wnt-amplified invasive state. Future work will need to determine whether YWHAG expression is enriched at invasive fronts, tumor buds or metastatic niches; whether YWHAG activity correlates with CTTN phosphorylation or cytoskeletal remodeling; and whether YWHAG-high tumors show enhanced dependence on Wnt-associated transcriptional programs or cytoskeletal vulnerabilities. Spatial proteomics and phospho-CTTN proximity assays could serve as initial validation tools.

### 4.3 YWHAE/14-3-3ϵ protein: EV secretion and Wnt-associated signaling

At the protein level, YWHAE/14-3-3ϵ has been implicated in extracellular-vesicle secretion and Wnt/β-catenin-related signaling in CRC. A 2025 study reported that YWHAE protein was highly expressed in CRC cells and tissues. YWHAE silencing inhibited CRC-cell proliferation and migration, increased apoptosis, reduced the expression of Wnt/β-catenin-related genes, impaired EV secretion and decreased β-catenin abundance in EV preparations derived from YWHAE-silenced cells (18).

These findings support an emerging protein-level YWHAE–EV/Wnt module. However, the current evidence does not identify a specific phosphorylation-dependent YWHAE client or demonstrate that YWHAE-dependent EV cargo is functionally transferred to recipient cells. Reduced EV secretion or altered EV-associated β-catenin abundance should therefore not be interpreted as direct evidence of functional intercellular Wnt signaling.

**Figure 2:**
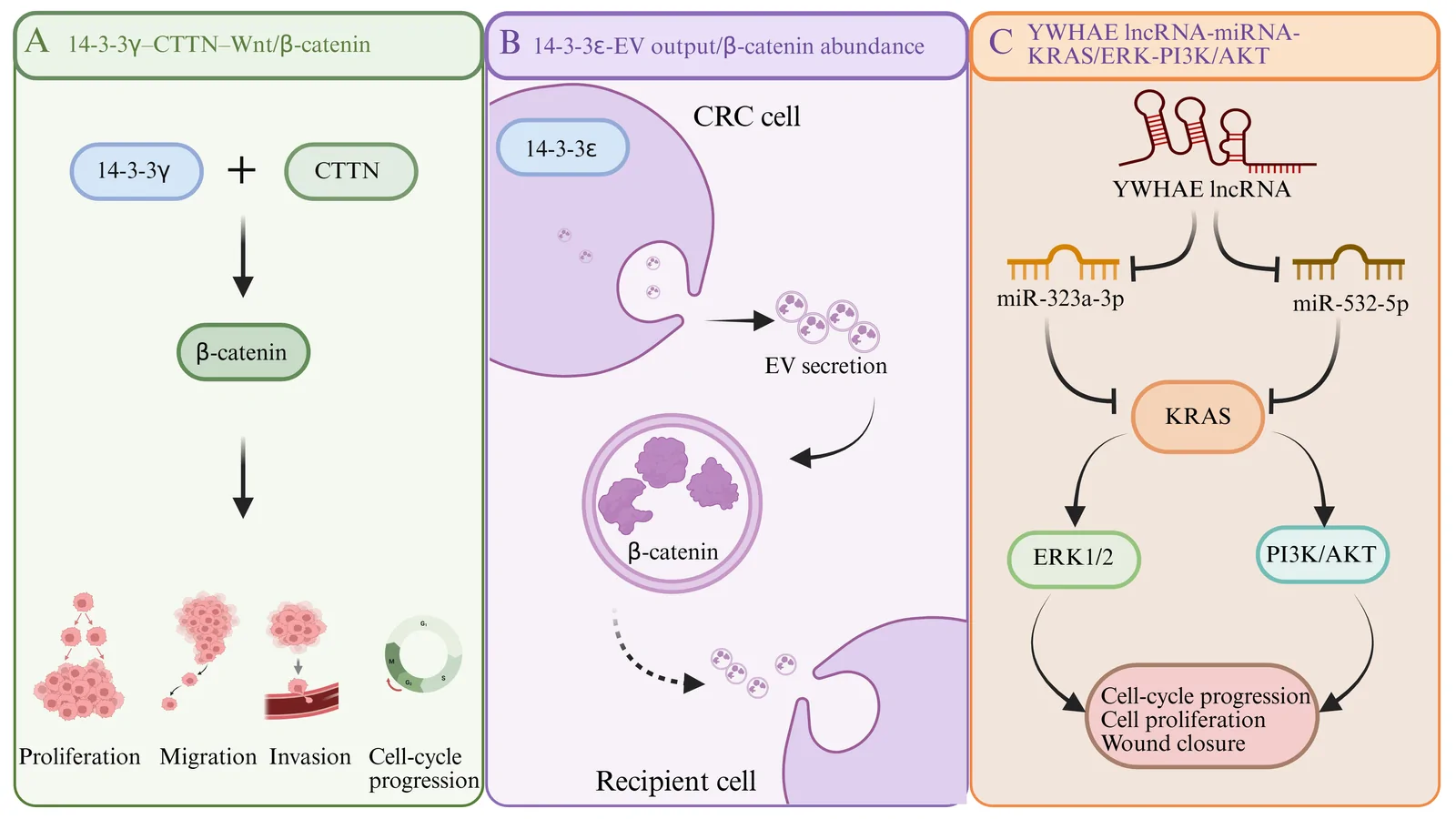
YWHAG and mechanistically distinct protein- and RNA-level YWHAE modules in CRC. **(A)** YWHAG–CTTN–Wnt/β-catenin. **(B)** YWHAE/14-3-3ϵ protein–EV/Wnt. **(C)** YWHAE lncRNA ceRNA signaling.

The EV component may nevertheless have translational relevance because EVs can carry proteins, nucleic acids, lipids and metabolites that reflect the cell of origin and may provide multicomponent liquid-biopsy readouts (45). EV-associated claims require standardized isolation, characterization and functional assays consistent with current MISEV recommendations (46). Future studies should determine whether YWHAE-associated EV β-catenin is detectable in plasma, stool or tumor interstitial fluid, whether it induces pathway activity in recipient cells, and whether it correlates with Wnt-active disease, metastatic progression or treatment response. The protein-level YWHAG–CTTN and YWHAE/14-3-3ϵ–EV/Wnt modules are illustrated in **Figures 2A, B**, respectively.

### 4.4 YWHAE lncRNA: a mechanistically distinct ceRNA module

YWHAE lncRNA is a non-coding transcript of the YWHAE gene and should be distinguished from the canonical YWHAE/14-3-3ϵ protein discussed above. Shared gene nomenclature does not establish a direct regulatory or functional relationship between the RNA transcript and the 14-3-3ϵ protein. No current CRC evidence demonstrates that YWHAE lncRNA regulates YWHAE protein abundance, dimerization, client binding or EV secretion.

YWHAE lncRNA was reported to be upregulated in CRC specimens and HCT116 cells and to interact with miR-323a-3p and miR-532-5p (19). Because these miRNAs target the KRAS 3′ untranslated region, YWHAE lncRNA overexpression increased KRAS expression and activated ERK1/2 and PI3K/AKT signaling. Functional assays further suggested that YWHAE lncRNA promoted cell-cycle progression, viability and migration in HCT116 cells (19).

These findings support a CRC-relevant ceRNA model but remain largely dependent on one principal cell-line system and a limited specimen set. This mechanism should therefore be interpreted as an independent RNA-regulatory module rather than as an extension of canonical 14-3-3ϵ protein-adaptor activity. Independent cohorts, additional CRC models, patient-derived organoids and *in vivo* validation are required before YWHAE lncRNA can be considered a clinically relevant biomarker or therapeutic dependency. The mechanistically distinct YWHAE lncRNA ceRNA module is shown separately in **Figure 2C**.

### 4.5 YWHAZ/14-3-3ζ: EMT and G2/M transition

YWHAZ/14-3-3ζ has broad cancer relevance and has been proposed as a therapeutic target in several malignancies (47, 48). In CRC, two mechanisms are particularly relevant: miRNA-mediated regulation of YWHAZ-driven EMT and TRIP13-YWHAZ-associated G2/M/EMT progression (20–23). The miR-1-3p-YWHAZ axis provides direct CRC evidence. miR-1-3p is downregulated in CRC, and restoration of miR-1-3p suppresses CRC cell proliferation and metastasis. YWHAZ was identified as a direct miR-1-3p target and mediated the associated EMT phenotype (20).

A second CRC-relevant mechanism involves the TRIP13-YWHAZ axis. TRIP13 is overexpressed in CRC and promotes proliferation, invasion, migration and tumor growth. Mechanistic work showed that TRIP13 interacts with YWHAZ and mediates G2/M transition and EMT (21). Additional studies have supported a metastasis-related role for TRIP13 in CRC, while the TRIP13-directed compound DCZ0415 has shown preclinical antitumor activity (22, 23). Therefore, the current CRC evidence supports YWHAZ as a component of EMT- and mitotic-transition networks rather than as a validated standalone therapeutic target. The most immediate translational value of this axis may lie in testing whether YWHAZ-associated states predict sensitivity to TRIP13-directed strategies, mitotic stress, EMT-state dependencies or combination approaches in patient-derived models.

### 4.6 YWHAB/14-3-3β: PIK3R2-dependent PI3K/AKT signaling

YWHAB/14-3-3β has historically received less attention in CRC than YWHAZ or SFN, but recent evidence supports its inclusion in isoform-resolved CRC networks. A 2023 study reported that YWHAB knockdown inhibited colon cancer cell proliferation, promoted G0/G1 arrest and induced apoptosis (24). Mechanistically, YWHAB bound PIK3R2, and YWHAB depletion reduced PIK3R2 expression and PI3K/AKT pathway activation. PIK3R2 overexpression reversed the effects of YWHAB knockdown, supporting a YWHAB-PIK3R2-PI3K/AKT survival module (24).

YWHAB has also been included in a chromosome 20q-associated CRC progression signature (25). Chromosome 20q amplification has been associated with distinct molecular features in metastatic CRC, although prognostic interpretation may vary by cohort and molecular context (26). Therefore, YWHAB is best regarded as an emerging survival-associated isoform and progression candidate, not as a standalone clinical biomarker. Its independent value requires testing in copy-number-aware cohorts, phospho-AKT readouts and patient-derived drug-response models.

### 4.7 YWHAH/14-3-3η: immune exhaustion and autophagy-associated invasion

YWHAH/14-3-3η is of particular interest because it connects tumor-intrinsic stress signaling with tumor-immune interactions. A 2024 study identified YWHAH as a potential therapeutic target for overcoming CD8+ T-cell exhaustion in CRC (27). The study linked YWHAH to NAT10-mediated N4-acetylcytidine (ac4C) modification, increased YWHAH stability, immune escape and poor clinical outcome. In CRC cell–CD8^+^ T-cell coculture systems, YWHAH was associated with impaired T-cell proliferation and increased exhaustion-related markers (27). Broader CRC work on precursor exhausted CD8+ T cells further supports the relevance of exhaustion-state mapping when evaluating YWHAH-linked immune programs (49).

A 2025 study further connected YWHAH to autophagy and MAPK/ERK signaling. YWHAH was highly expressed in CRC tissues and associated with poor differentiation, advanced TNM stage, lymph-node metastasis, vascular invasion and poor prognosis. Functionally, YWHAH regulated autophagy through the MAPK/ERK pathway and promoted CRC migration and invasion (28). These data do not establish a universal pro- or anti-autophagic role for YWHAH. Rather, they support a context-dependent role for YWHAH in MAPK/ERK-dependent autophagy-associated invasion.

This module is translationally relevant because it links immune evasion, autophagy and therapy response. Autophagy has context-dependent roles in CRC, supporting survival, metastasis or treatment resistance in some settings while contributing to cell death in others (50). Thus, YWHAH warrants evaluation in immune-competent models, tumor-organoid and CD8+ T-cell cocultures, spatial immune profiling and therapy-response cohorts. Key questions include whether YWHAH-associated autophagy modulation affects antigen presentation, PD-L1 expression, EV release, CD8+ T-cell exhaustion or resistance to immune-checkpoint blockade.

### 4.8 SFN/14-3-3σ: context-dependent comparator isoform

SFN/14-3-3σ functions as a useful comparator isoform because it demonstrates that both YWHA identity and cellular context must be considered. SFN was originally identified as a p53-regulated G2/M checkpoint effector and is required to prevent mitotic catastrophe after DNA damage (29, 30). In intestinal tumorigenesis, 14-3-3σ can function as a tumor suppressor, and loss of SFN has been linked to increased adenoma burden and epithelial destabilization (31).

Additional CRC mechanisms reinforce the tumor-suppressive side of SFN. The 14-3-3σ-NEDD4L-HIF-1α axis promotes HIF-1α ubiquitination and degradation and suppresses hypoxia-induced metastasis and angiogenesis (32). The TGF-β/SMAD4/14-3-3σ/TFEB axis promotes mesenchymal-epithelial transition and inhibits autophagy in CRC (34). These findings suggest that SFN can preserve epithelial identity, restrain hypoxia-associated progression and modulate autophagy-related plasticity in selected genetic contexts.

**Figure 3:**
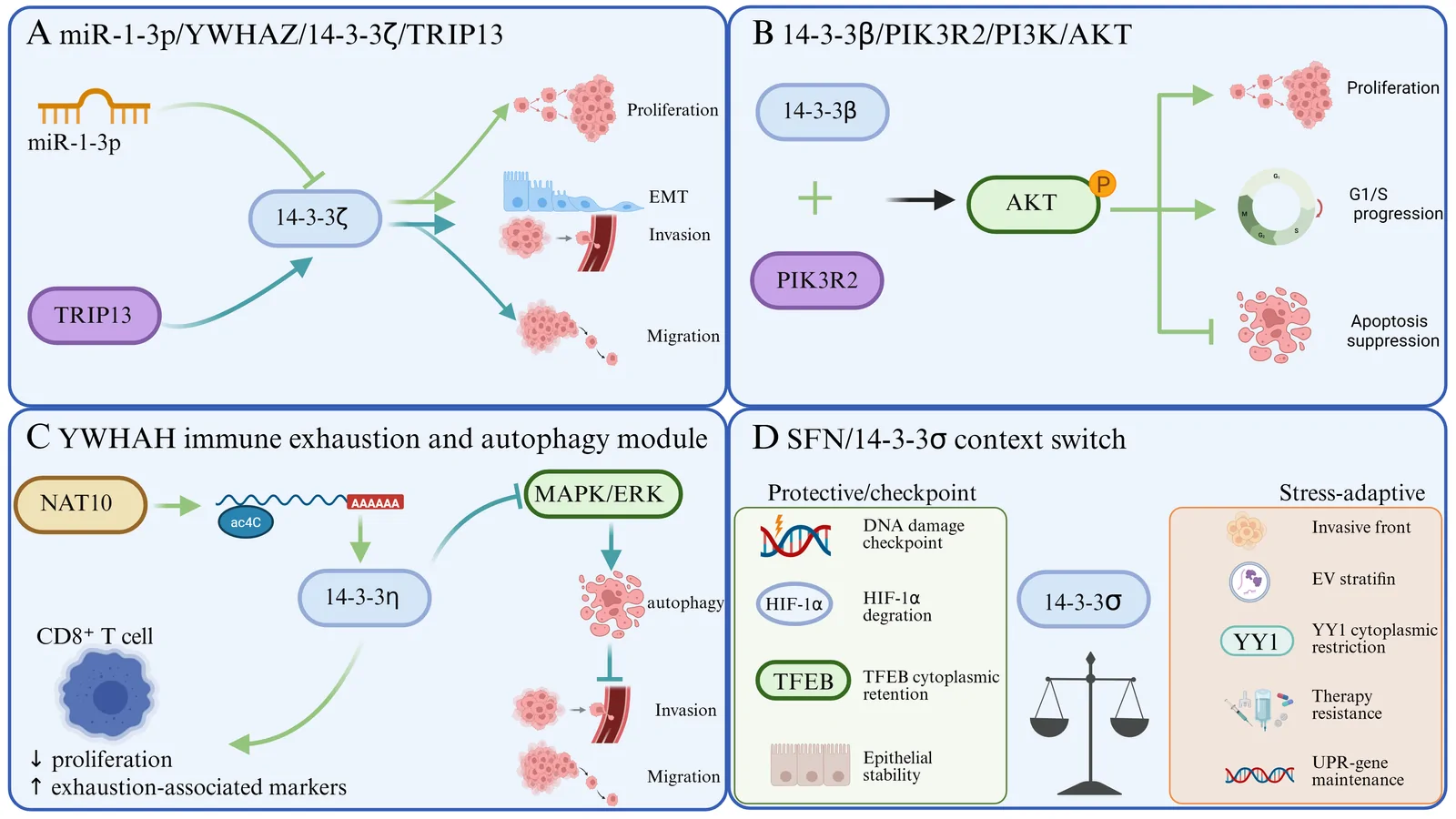
Selected non-Wnt YWHA modules in CRC. **(A)** YWHAZ-associated EMT and G2/M regulation. **(B)** YWHAB–PIK3R2–PI3K/AKT. **(C)** Independently reported YWHAH immune-exhaustion and autophagy modules. **(D)** Context-dependent SFN networks.

However, SFN is not uniformly suppressive. Earlier clinical studies reported SFN overexpression in poor-prognosis CRC subsets (35), and invasive-front studies linked 14-3-3σ expression to tumor budding and tenascin-C-dependent migration (36). Serum EV-associated stratifin has also been proposed as a potential biomarker of perineural invasion in stage II CRC (37). More recently, SFN was shown to restrict YY1 to the cytoplasm, promote UPR signaling and support therapy resistance (33). Together, these findings illustrate that a single 14-3-3 isoform can exert divergent functions depending on genetic background, spatial compartment and stress state. A practical way to interpret SFN in CRC is as a context-switching isoform rather than as a fixed tumor suppressor or oncogene. In p53-competent, epithelial or DNA-damage checkpoint contexts, SFN may help preserve genomic control and epithelial stability. In contrast, at invasive fronts or under hypoxic, extracellular-matrix-rich or therapy-induced stress, SFN-associated complexes may support migration, UPR signaling or treatment adaptation. Future studies should therefore stratify SFN analyses by p53 status, SMAD4/TGF-β context, hypoxia, invasive-front localization, UPR activation and treatment exposure. Non-Wnt modules involving YWHAZ, YWHAB, YWHAH and SFN are summarized in **Figure 3**.

### 4.9 YWHAQ/14-3-3θ: a defined evidence gap in CRC

YWHAQ/14-3-3θ, also termed 14-3-3τ, is one of the seven canonical mammalian 14-3-3 isoforms (14, 16). Within the CRC literature identified by our search, we found no study that established a YWHAQ-specific phosphorylated client, direct molecular mechanism, causal tumor phenotype, or validated association with prognosis or treatment response. YWHAQ should therefore not currently be classified as oncogenic, tumor-suppressive, biomarker-relevant or therapeutically actionable in CRC. Accordingly, YWHAQ-related evidence was assigned to Category D.

This absence of CRC-specific mechanistic evidence defines a discovery priority rather than a biological conclusion. Future studies should include YWHAQ in isoform-resolved protein quantification, endogenous interactomics, phosphoproteomics, proximity-labeling and loss-of-function/rescue experiments across molecularly annotated CRC models. Any candidate YWHAQ module should subsequently be confirmed using isoform-specific reagents, direct complex assays, patient-derived models and *in vivo* validation before functional or translational claims are made.

## 5 Integration with CRC molecular subtypes and clinical phenotypes

A central translational question is how YWHA isoform–client modules map onto clinically relevant CRC states. TCGA established major genomic and epigenomic features of colon and rectal cancer, including hypermutated and non-hypermutated disease, recurrent mutations in APC, TP53, KRAS, PIK3CA and SMAD4, and frequent copy-number alterations (3). The consensus molecular subtype (CMS) taxonomy further separated CRC into CMS1 immune/MSI, CMS2 canonical Wnt/MYC, CMS3 metabolic and CMS4 mesenchymal subtypes (4). The clinical value of CMS classification has been systematically reviewed and appears context-dependent across disease stage and treatment setting (51). Recent metastatic CRC data also suggest that CMS assignment may expand biomarker-directed therapeutic interpretation in CMS1 and CMS2 tumors (52). Proteogenomic profiling demonstrated that protein and pathway-level information can reveal therapeutic opportunities not evident from mRNA data alone (5).

At present, YWHA isoform–client modules should be mapped onto CRC molecular subtypes as hypothesis-generating overlays, not as validated CMS or MSI biomarkers. With this limitation in mind, YWHAG and YWHAE may be most relevant to Wnt-high, epithelial or EV-secreting states; YWHAZ/TRIP13 to proliferative and EMT-associated programs; YWHAB to PI3K/AKT survival or chromosome-20q-associated progression; YWHAH to immune-exhausted, autophagy-associated or stress-adapted compartments; and SFN to genotype- and stress-dependent checkpoint or resistance states.

Clinical integration also needs to account for microsatellite status. Immune-checkpoint blockade has transformed treatment of dMMR/MSI-H metastatic CRC, as shown by PD-1 blockade studies and KEYNOTE-177 (53–55). Neoadjuvant PD-1 blockade has also shown striking activity in mismatch repair-deficient locally advanced rectal cancer (56). However, most metastatic CRCs are microsatellite stable and respond poorly to checkpoint blockade, motivating efforts to identify immune-excluded or immune-exhausted states that may be therapeutically remodeled (57). The YWHAH-NAT10/ac4C and YWHAH-autophagy axes provide hypothesis-generating entry points for studying such states, but clinical implementation requires spatial immune profiling and therapeutic validation.

Anti-EGFR therapy and acquired resistance provide another clinically relevant context. Resistance to EGFR blockade in metastatic CRC involves intratumor heterogeneity, convergent evolution and emergence of RAS, BRAF, EGFR, HER2, MET or downstream pathway alterations (58). YWHAE lncRNA-mediated KRAS/ERK and PI3K/AKT activation and YWHAB-PIK3R2-dependent PI3K/AKT signaling are therefore relevant as upstream adaptor or RNA-mediated mechanisms that may influence pathway dependency. At present, they are best regarded as testable mechanisms rather than clinical predictors.

Single-cell and spatial studies further support the need for compartment-aware interpretation. Lineage-dependent cancer-cell programs influence immune landscapes in CRC (59), FAP^+^ fibroblasts and SPP1^+^ macrophages interact to define immunosuppressive stromal niches (60), and integrative single-cell analysis has identified patient strata with distinct immune evasion mechanisms (61). Spatial proteomic studies have begun to reveal microenvironment heterogeneity and immunotherapy-associated protein patterns in CRC (62, 63). These platforms are well suited for mapping YWHA isoform–client complexes at invasive fronts, tumor-stroma boundaries, immune-excluded regions, metastatic niches and therapy-resistant compartments.

Clinical sampling is also important when interpreting YWHA biology. Primary tumors, lymph-node metastases, liver metastases, peritoneal implants and post-treatment lesions may differ in stromal content, immune infiltration, copy-number architecture, EV release and stress-response programs. Left-sided and right-sided tumors also differ in molecular composition and treatment selection. YWHA biomarker development will require approaches beyond unstratified bulk tumor tissue alone. A robust clinical-development plan would compare matched normal mucosa, primary tumors, metastatic lesions and treatment-resistant samples, while annotating MSI status, RAS/BRAF/PIK3CA genotype, SMAD4 status, sidedness, prior therapy and metastatic site. These considerations are consistent with clinical sequencing and sidedness studies showing that metastatic CRC is molecularly heterogeneous and that primary tumor location and metastatic site can influence biomarker interpretation (64–66).

## 6 Translational hypotheses and biomarker opportunities

### 6.1 Biomarker stratification

A YWHA-centered biomarker strategy in CRC should focus on active molecular modules rather than isolated gene or protein abundance. Informative assays should distinguish isoform expression, availability of the proposed client or regulatory partner, complex formation or phosphorylation-dependent activity, and localization within a relevant tumor compartment. Candidate readouts include YWHAG–CTTN, YWHAE/14-3-3ϵ protein-associated EV β-catenin, YWHAE lncRNA-associated KRAS/ERK and PI3K/AKT activity, TRIP13–YWHAZ, YWHAB–PIK3R2, NAT10/ac4C–YWHAH, YWHAH-associated autophagy and context-specific SFN–client complexes.

Because these candidate modules differ in evidence maturity, their translation requires sequential validation from discovery and analytical verification to mechanistic, context-specific and clinical assessment. This stage-gated framework is summarized in **Supplementary Table 1**. Diagnostic and prognostic studies should follow appropriate reporting frameworks, including REMARK and STARD, respectively (67, 68), while module-specific applications and experimental priorities are provided in **Supplementary Table 2**.

### 6.2 Clinical treatment contexts and candidate response biomarkers

Clinically established CRC therapies provide settings in which YWHA modules may be evaluated as exploratory response or resistance biomarkers; they should not be interpreted as direct YWHA-targeted treatments. In anti-EGFR-treated disease, YWHAE lncRNA-associated KRAS/ERK and PI3K/AKT activity and YWHAB–PIK3R2 signaling may indicate persistent downstream pathway activation (19, 24). In chemotherapy-treated disease, SFN–YY1/UPR may represent a stress-adaptive state (33), whereas NAT10/ac4C–YWHAH may be relevant to immune-exhausted tumors considered for immune-checkpoint blockade (27).

Other modules, including YWHAG–CTTN, YWHAE/14-3-3ϵ protein–EV/Wnt and TRIP13–YWHAZ, may identify Wnt-active, EV-communicating, invasive or mitotic-stress states (17, 18, 21). However, no YWHA module is currently a clinically validated treatment-selection biomarker. Their value should therefore be tested in treatment-annotated cohorts and assessed for added information beyond established molecular and clinicopathological variables.

Direct intervention remains less mature. TRIP13-directed compounds, selective YWHA–client modulation, 14-3-3 stabilizers and molecular-glue approaches remain preclinical or proof-of-concept strategies in CRC (23, 39, 69). The immediate translational priority is therefore to evaluate YWHA modules as context-specific biomarkers within established treatment settings rather than to imply that direct YWHA-targeted therapy is clinically available.

### 6.3 EV and liquid-biopsy opportunities

YWHAE/14-3-3ϵ protein and SFN support the investigation of EV-associated biomarkers. YWHAE silencing reduces EV secretion and EV-associated β-catenin, whereas serum EV-associated SFN has been linked to perineural invasion in stage II CRC (18, 37). These observations do not yet establish functional cargo transfer, tumor-cell origin or causal involvement in the associated phenotype.

Further development requires standardized EV isolation and characterization, confirmation that the candidate cargo is vesicle-associated, recipient-cell transfer assays and independent clinical validation consistent with MISEV recommendations (45, 46, 70). EV-associated YWHAE and SFN should therefore be regarded as liquid-biopsy candidates rather than validated clinical assays.

### 6.4 Preclinical interaction-guided drug development

Pan-family 14-3-3 inhibition would risk disrupting essential and protective functions (13). A more plausible strategy is selective modulation of disease-relevant interactions, including YWHAG–CTTN, TRIP13–YWHAZ, YWHAB–PIK3R2 and defined SFN- or YWHAH-associated complexes. Although 14-3-3 stabilizers and molecular glues demonstrate the chemical feasibility of interaction modulation, most remain tool compounds or proof-of-concept agents, and none has established clinical efficacy in CRC (39, 40, 69). DCZ0415 should likewise be described as a preclinical TRIP13-directed compound rather than a direct YWHAZ inhibitor (23).

Candidate interactions should advance only when they show tumor-context selectivity, nonredundant mechanistic dependency, measurable target engagement and an acceptable therapeutic window. This is particularly important for SFN, because modulation of protective and stress-adaptive SFN complexes may produce opposite biological effects.

## 7 Experimental models for context-specific validation

Experimental systems should be selected according to the biological question and the stage of validation. Public transcriptomic, proteomic and phosphoproteomic datasets, including TCGA and CPTAC, can nominate candidate isoform–client modules (3, 5), whereas single-cell and spatial datasets can localize these modules to epithelial, stromal, immune, invasive-front or metastatic compartments (59–63). These discovery platforms are useful for prioritization but cannot establish molecular causality.

Patient-derived tumor organoids provide a more clinically relevant setting for testing whether YWHA-associated dependencies are maintained within patient-specific genomic and phenotypic contexts. CRC organoid biobanks preserve important features of the original tumors and can support functional drug-response assessment (71–74). Depending on the proposed mechanism, organoids may be combined with genetic perturbation, rescue experiments, invasion systems, extracellular-matrix remodeling or treatment-response assays. *In vivo* models remain necessary when the hypothesis concerns tumor growth, metastatic dissemination, immune regulation or treatment adaptation.

Model selection should also reflect the specific YWHA module. YWHAH-associated immune-exhaustion hypotheses require tumor–CD8+ T-cell cocultures, immune-competent models and spatial profiling of exhaustion markers. YWHAE/14-3-3ϵ protein-associated EV mechanisms require standardized vesicle characterization, confirmation of cargo localization, recipient-cell uptake and functional transfer assays. Modules associated with invasion or metastasis should be evaluated in orthotopic, metastatic or spatially resolved models rather than proliferation assays alone. Interaction-dependent mechanisms should be confirmed using co-immunoprecipitation, proximity ligation, targeted phosphoproteomics, proximity labeling or interaction- and phosphosite-deficient mutants (75). Model selection should therefore be guided by the proposed molecular mechanism, relevant tumor compartment and intended translational question.

## 8 Discussion: challenges, controversies and future directions

Current evidence remains limited by its reliance on CRC cell lines, database analyses and relatively small or single-center cohorts. Total YWHA mRNA or protein abundance may not reflect active signaling because function also depends on dimer composition, phosphorylation-dependent client binding and subcellular localization (14, 16). Most studies perturb a single isoform without systematically testing compensation by other family members, making strict isoform specificity difficult to establish.

Mechanisms sharing the same isoform designation should not automatically be combined into a single pathway. The YWHAE/14-3-3ϵ protein–EV/Wnt module and the YWHAE lncRNA ceRNA module are mechanistically distinct (18, 19). Similarly, the miR-1-3p–YWHAZ and TRIP13–YWHAZ mechanisms were established independently (20, 21). The immune-exhaustion and autophagy-associated YWHAH modules also arose from different experimental settings (27, 28). Similar phenotypic outputs therefore do not establish a shared linear pathway.

Autophagy and SFN illustrate particularly strong context dependence. YWHAH-associated autophagy has been linked to invasion, whereas the TGF-β/SMAD4–SFN–TFEB axis suppresses autophagy and promotes epithelial features (28, 34). SFN can support epithelial stability and restrain hypoxia-associated progression in selected contexts (31, 32). In contrast, other studies have linked SFN to invasive-front migration or UPR-associated treatment adaptation (33, 36). These differences may reflect genotype, client identity, spatial compartment, treatment exposure or model design.

EV-associated and therapeutic claims also require caution. Altered YWHAE-associated EV secretion or cargo does not establish functional transfer to recipient cells (18), whereas the clinical association of EV-associated SFN with perineural invasion does not demonstrate causality (37). Direct 14-3-3 interaction modulators and molecular glues remain preclinical or proof-of-concept approaches (39, 76), and DCZ0415 is a preclinical TRIP13-directed compound rather than a direct YWHAZ inhibitor (23). Future studies should prioritize isoform-specific interaction assays, rescue experiments, spatially resolved samples, patient-derived and immune-competent models, standardized EV-transfer studies and treatment-annotated cohorts.

## 9 Conclusions

YWHA/14-3-3 proteins in CRC are best interpreted as isoform–client-context networks rather than as a homogeneous adaptor family. The available evidence supports distinct modules centered on YWHAG–CTTN–Wnt/β-catenin, YWHAE–EV/Wnt and YWHAE lncRNA–KRAS/ERK/PI3K/AKT, YWHAZ–miRNA/TRIP13–EMT/G2/M, YWHAB–PIK3R2–PI3K/AKT, YWHAH–NAT10/ac4C and MAPK/ERK–autophagy, and context-dependent SFN-client networks.

The clinically relevant question is not whether 14-3-3 proteins are oncogenic or tumor suppressive in general, but which isoform, which phosphorylated client, which tumor compartment and which stress state are involved. If validated through phosphoproteomics, interactomics, spatial pathology, EV profiling, organoid-drug response models and clinical cohorts, this framework could support biomarker interpretation and interaction-guided therapeutic strategies. The translational opportunity is to move from descriptive YWHA expression toward testable, context-defined isoform–client mechanisms that can be validated in spatially resolved tissues, extracellular-vesicle assays, patient-derived models and therapy-response cohorts.
