# Supplementary material for: Isoform-resolved 14-3-3/YWHA networks in colorectal cancer: phospho-adaptor mechanisms, biomarker contexts and therapeutic opportunities
Source: Front Oncol. 2026 Aug 19;16:1906093. doi: 10.3389/fonc.2026.1906093 (PMC13536284; doi:10.3389/fonc.2026.1906093)
Supplement: Supplementary file 1 [file Table1.docx]

Supplementary Table 1. Stage-gated translational validation roadmap for YWHA isoform–client modules in CRC.

| Stage | Core question | Minimum evidence required | Representative approaches | Advancement criterion |
| --- | --- | --- | --- | --- |
| 1. Candidate discovery | Is the proposed YWHA module reproducibly associated with a defined CRC state? | Reproducible isoform, client, RNA or EV signal in CRC datasets or specimens, with annotation of molecular subtype, compartment or treatment context. | Transcriptomics, proteomics, phosphoproteomics, single-cell analysis, spatial profiling and clinically annotated cohorts. | The candidate is reproducible in an independent dataset, cohort or sample type and is linked to a clearly defined CRC context. |
| 2. Analytical and complex validation | Can the active module be detected specifically and reproducibly? | Isoform discrimination, assay specificity and evidence that the measured signal reflects the proposed complex or molecular activity rather than total expression alone. | Isoform-specific immunoblotting or IHC, targeted proteomics, co-immunoprecipitation, proximity ligation, phosphosite assays and standardized EV analysis. | The assay distinguishes the relevant isoform and detects the proposed client interaction, phosphorylation state or EV-associated signal with acceptable reproducibility. |
| 3. Mechanistic causality | Does the module causally regulate the proposed phenotype? | Perturbation of the isoform and client or regulatory partner, together with rescue, epistasis or phosphosite-dependent validation. | Knockdown, knockout, re-expression, interaction-deficient mutants, phosphosite mutants and orthogonal functional assays. | The phenotype depends on the defined isoform–client or RNA-regulatory mechanism and is restored or reversed by an appropriate rescue experiment. |
| 4. Context and model validation | Is the dependency maintained in clinically relevant CRC states and compartments? | Confirmation in molecularly annotated patient-derived or in vivo models and localization to a biologically relevant compartment. | Patient-derived tumor organoids, xenografts, immune-competent models, tumor–immune cocultures, metastatic models, spatial pathology and EV-transfer assays. | The module remains active and functionally relevant in the intended genotype, tumor compartment, metastatic site or treatment state. |
| 5. Clinical validation and utility | Does the module provide clinically meaningful information beyond existing variables? | Independent clinical validation with predefined endpoints and comparison with established clinicopathological and molecular factors. | Treatment-annotated tissue or liquid-biopsy cohorts, longitudinal sampling and prospective or retrospective validation following appropriate reporting standards. | The biomarker shows reproducible association with diagnosis, prognosis, metastasis or treatment response and adds value beyond established clinical markers. |

Note: The stages define a progressive validation framework; individual studies may address more than one stage.

Supplementary Table 2. Module-specific candidate applications and validation priorities for YWHA-related mechanisms in CRC.

| **Module** | **Candidate biomarker use** | **Therapeutic or biological hypothesis** | **Priority validation** | **Current evidence status** |
| --- | --- | --- | --- | --- |
| YWHAG–CTTN–Wnt/β-catenin | Invasive-state or Wnt-amplification marker | YWHAG–CTTN may identify CRC cells with enhanced Wnt-associated cytoskeletal remodeling and invasive behavior | Spatial IHC or IF, phospho-CTTN/proximity assays, PDTO invasion assays, in vivo validation | Mechanistically supported; spatial and in vivo validation needed |
| YWHAE–EV/Wnt | EV-associated Wnt-communication marker | YWHAE may support EV secretion and EV-associated β-catenin/Wnt output | MISEV-compliant EV isolation, EV cargo quantification, recipient-cell transfer assays, plasma/stool validation | Discovery-to-mechanistic; EV functional transfer remains to be validated |
| YWHAE lncRNA–KRAS/ERK/PI3K/AKT | RNA-layer pathway-activation marker | YWHAE lncRNA may promote MAPK and PI3K pathway activation through competing endogenous RNA mechanisms | Independent cohort validation, miRNA–KRAS reporter assays, pathway-inhibitor response testing, PDTO validation | Hypothesis-generating; clinical breadth unclear |
| TRIP13–YWHAZ | EMT/cell-cycle state marker | TRIP13–YWHAZ-associated states may create vulnerability to mitotic stress, EMT-state dependencies or TRIP13-directed strategies | TRIP13–YWHAZ proximity assays, mitotic-stress assays, EMT-state profiling, DCZ0415 or TRIP13-inhibition sensitivity testing | Mechanistically supported; YWHAZ-specific therapeutic dependency unproven |
| YWHAB–PIK3R2–PI3K/AKT | PI3K/AKT survival-dependency marker | YWHAB may support CRC cell survival through PIK3R2-dependent PI3K/AKT signaling | PIK3R2 rescue assays, phospho-AKT readouts, copy-number-aware cohort analysis, inhibitor-response testing in PDTOs | Early mechanistic evidence; patient and in vivo validation needed |
| YWHAB–20q progression signature | Progression or copy-number-context marker | YWHAB may contribute to chromosome-20q-associated CRC progression programs | Copy-number-stratified cohorts, protein-level validation, integration with metastatic-site and treatment-response data | Association-level evidence; independent biomarker value unvalidated |
| NAT10/ac4C–YWHAH | Immune-exhaustion or epitranscriptomic-state marker | NAT10/ac4C-dependent YWHAH regulation may contribute to CD8+ T-cell exhaustion and immune escape | ac4C mapping, YWHAH mRNA/protein stability assays, tumor–CD8+ T-cell coculture, spatial exhaustion-marker profiling | Mechanistically supported; immune-competent and therapy-response validation needed |
| YWHAH–MAPK/ERK–autophagy | Autophagy-associated invasion marker | YWHAH may promote migration and invasion through MAPK/ERK-dependent autophagy regulation | Autophagy-flux assays, ERK-inhibition rescue, invasion assays, immune coculture and PDTO validation | Early-to-mechanistic evidence; autophagy direction and clinical utility require clarification |
| SFN–NEDD4L–HIF-1α / SFN–TFEB | Context-dependent epithelial or hypoxia/autophagy marker | SFN-associated complexes may restrain hypoxia-driven progression or modulate autophagy depending on genetic context | Stratification by p53, SMAD4/TGF-β status, hypoxia markers and epithelial-state readouts | Context-dependent mechanistic evidence; stratified validation required |
| SFN–YY1/UPR | Therapy-stress or resistance-state marker | SFN may support unfolded-protein-response signaling and treatment adaptation in selected CRC contexts | YY1 localization assays, UPR readouts, chemotherapy or targeted-therapy stress models, resistant PDTO cohorts | Mechanistically supported in stress contexts; clinical predictive value unvalidated |
| YWHAQ-associated modules | No established biomarker use; discovery-stage candidate | No CRC-specific biological or therapeutic role can currently be assigned | Isoform-resolved proteomics, phosphoproteomics, endogenous interactomics, proximity labeling and functional perturbation | Evidence gap; discovery-level priority |
